# Multiparametric MRI characteristics for differentiating primary cancer origin in brain metastases

**DOI:** 10.1186/s12880-025-01925-5

**Published:** 2025-10-15

**Authors:** Ali Salbas, Mehmet Coskun, Yusuf Kenan Cetinoglu, Merve Horoz, Murat Yogurtcu, Anil Huvez, Mustafa Fazil Gelal

**Affiliations:** 1 Department of Radiology, Ataturk Training and Research Hospital, Izmir Katip Celebi University, Izmir, 35150 Turkey; 2 Department of Radiology, University of Health Sciences Türkiye Dr. Behçet Uz Children’s Diseases and Surgery Training and Research Hospital, Izmir, 35100 Turkey; 3 Department of Radiology, Izmir Bakircay University, Izmir, 35665 Turkey; 4 Department of Radiology, Tire State Hospital, Izmir, 35900 Turkey; 5 Department of Radiology, Manisa Merkezefendi State Hospital, Manisa, 45120 Turkey

**Keywords:** ADC, Brain metastases, Breast cancer, ITSS, Lung cancer, Malignant melanoma, Necrosis, Perilesional edema, Primary tumor origin, rCBV

## Abstract

**Background:**

Accurate identification of primary cancer origin in brain metastases (BMs) is crucial for diagnosis and treatment planning. Identification is challenging, particularly in unknown primary cases. Multiparametric Magnetic Resonance Imaging (MRI) offers non-invasive imaging characteristics as diagnostic clues. This study aimed to investigate the relationship between multiparametric MRI features of BMs and their primary tumor origin.

**Methods:**

This retrospective study included 125 patients with intra-axial brain metastases, classified as breast cancer, small cell lung cancer (SCLC), non-small cell lung cancer (NSCLC), malignant melanoma, and other cancers. Multiparametric MRI, including T2-FLAIR, contrast-enhanced 3D T1-weighted imaging, apparent diffusion coefficient (ADC) maps, susceptibility-weighted imaging (SWI), and cerebral blood volume (CBV) maps, was performed. Lesion volume, necrotic volume, necrosis ratio, peritumoral edema volume, and edema ratio were measured. 3D Slicer^®^ software was used for semi-automated volumetric measurements. Quantitative ADC and rCBV values, intratumoral susceptibility signal (ITSS) scores, and metastasis number and location were analyzed to differentiate primary tumor origins.

**Results:**

291 metastatic lesions from 125 patients were analyzed. NSCLC metastases showed significantly higher necrosis ratios than breast (*p* < 0.001) and SCLC (*p* = 0.025) metastases. Malignant melanoma metastases exhibited significantly higher edema ratios than breast (*p* = 0.001) and SCLC (*p* = 0.004) metastases. NSCLC metastases also showed a significantly higher edema ratio than breast (*p* = 0.011) metastases. Mean ADC values were significantly higher in NSCLC metastases compared to all other groups, with an optimal cut-off of ≥ 0.905 mm²/s (AUC: 0.775). Malignant melanoma and SCLC metastases had significantly higher rCBV values than breast cancer metastases. Malignant melanoma metastases consistently showed the highest ITSS scores among all groups, with an optimal cut-off of ≥ 1 (AUC: 0.769). Furthermore, multiple metastases ≤ 0.5 cm³ were significantly associated with breast cancer (*p* = 0.041). Infratentorial metastases were more prevalent in breast cancer (OR = 2.490, *p* = 0.001).

**Conclusion:**

Brain metastases exhibit distinct multiparametric MRI characteristics by primary cancer origin. Breast cancer metastases tend to be smaller, multiple, and infratentorial. NSCLC metastases show greater necrosis and higher ADC values, while melanoma metastases demonstrate higher intratumoral susceptibility. These features offer valuable diagnostic clues for differentiating primary cancer types in patients with brain metastases.

**Clinical trial number:**

Not applicable.

## Introduction

Brain metastases (BMs) are the most common intracranial tumors [1–4]. They are observed ten times more frequently than primary brain malignant tumors [3]. The most common primary sources of BMs are lung cancer, breast cancer, and malignant melanoma, accounting for two-thirds of all intracranial metastases [4]. One in five patients diagnosed with cancer develops BMs (4). Advances in cancer treatment and prolonged patient survival have contributed to an increased incidence of BMs [2].

The primary cancer cannot be identified in 15% of cases with BMs [5]. Cancers of unknown primary origin are the seventh to eighth most common malignancies [6]. In cases of BMs, lung cancer origin—particularly the small cell subtype—significantly reduces survival [7]. The radiological identification of BMs along with the prediction of their origin facilitates the detection of primary cancer and prognostic assessment.

Recent studies in radiomics and deep learning show promising potential in distinguishing the primary origin of brain metastases, differentiating glioblastoma from solitary brain metastases, and addressing other complex neuroimaging scenarios [8–11]. While these techniques are increasingly being adopted, their integration into daily clinical workflows across diverse institutions, particularly outside of specialized centers, may still be limited. Therefore, conventional multiparametric magnetic resonance imaging (MRI) remains the most accessible and clinically practical tool for evaluating brain metastases. Most previous MRI-based studies have focused on differentiating brain metastases from glial tumors [12]. However, studies aiming to distinguish brain metastases based on their primary tumor origin have remained limited and have mostly compared only a small number of metastasis types with each other. To the best of our knowledge, this is the first study in which all these parameters (tumor volume, necrosis and edema ratios, ADC, rCBV, and ITSS) have been evaluated together for differentiating primary tumor types of brain metastases. In this study, we aimed to investigate whether multiparametric MRI features can help predict the primary origin of brain metastases at the time of diagnosis and thereby contribute to filling a gap in the current imaging literature.

## Materials and methods

### Study population

Local ethics committee approval was obtained for this retrospective study (2024-SAEK-0129, Decision No: 0112). This study included patients with intra-axial BM with known primary origin between 2019 and 2023. The patients underwent brain MRI due to intracranial screening or the development of cranial symptoms. The initial brain MRI performed at the time of brain metastasis diagnosis, prior to any brain metastasis–directed treatment, was evaluated. Histopathological confirmation of metastatic lesions was not available in all cases; diagnoses were based on clinical and radiological assessment in conjunction with primary tumor evaluation and follow-up data.

Lesions with major hemorrhages causing substantial distortions and artifacts (*n* = 8) were excluded. Patients whose initial MRI lacked contrast-enhanced three-dimensional T1-weighted (3D T1W) images or lacked both fluid-attenuated inversion recovery (FLAIR) and T2-weighted (T2W) sequences were also excluded (*n* = 7). Additionally, patients who had received chemotherapy, intracranial radiotherapy, or anti-edema treatment—including corticosteroids—prior to MRI acquisition (*n* = 14) were excluded, as these therapies can alter lesion size, edema, and perfusion characteristics, potentially confounding imaging-based analyses. Moreover, cases with an unknown primary origin (*n* = 2) and one patient with two concurrent primary malignancies—detected during systematic screening—were excluded due to the uncertainty regarding the origin of the brain metastasis. For all included cases, systematic screening methods such as thoracoabdominal computed tomography (CT), endoscopy-colonoscopy, and positron emission computed tomography (PET-CT) confirmed the absence of a synchronous malignancy. As a result, the study cohort consisted of 125 patients with 291 metastatic lesions.

### Magnetic resonance imaging acquisition

MRI examinations were acquired on two different 1.5 Tesla scanners: MAGNETOM (Siemens Healthcare, Erlangen, Germany) and Optima 360 (General Electric, Fairfield, USA). Detailed acquisition parameters for each scanner are presented in Tables 1 and 2.

Perfusion imaging was available only on the first scanner and was performed in 89 patients (71.2%) using the dynamic susceptibility contrast (DSC) technique. A total dose of 0.1 mmol/kg gadolinium-based contrast agent was administered. Approximately half of the dose was given as a saturation bolus, followed five minutes later by the remaining dose via an infusion pump at a rate of 5 ml/sec, followed by a 20 ml saline flush. Imaging was performed using T2*-weighted gradient-echo echo-planar (GRE-EPI) sequences with a temporal resolution of 2 s.

**Table 1:** Siemens Magnetom 1.5 Tesla MRI sequence parameters

Parameter	T2W	T2-FLAIR	DWI	Contrast-Enhanced T1W	T2* (DSC Perfusion)	SWI
TE (ms)	99	86	60	4,86	30	40
TR (ms)	4000	8000	4800	13	3040	49
TI (ms)		2371		-	-	
Slice Thickness (mm)	5	5	5	1	3	2.6
Slice Gap (mm)	1.5	1.5	1.5	1.5	3.9	1
Field of View (FOV) (mm)	230	230	230	230	220	230
b-value (s/mm²)	-	-	1000	-	-	-

**Table 2:** GE Optima 360 1.5 Tesla MRI sequence parameters

Parameter	T2W	T2 FLAIR	DWI	Contrast-Enhanced T1W	SWAN
TE (ms)	84	122	103.1	3.9	49.4
TR (ms)	4222	9000	5656	10	76.3
TI (ms)	-	2200	-	-	-
Slice Thickness (mm)	5.5	5.5	5.5	1.4	4
Slice Gap (mm)	1.6	1.6	1.6	1.6	1
Field of View (FOV) (mm)	220	220	220	256	250
b-value (s/mm²)	-	-	1000	-	-

Note: In the GE scanner, SWAN (Susceptibility Weighted Angiography) was used as the equivalent of SWI

### Multiparametric MRI analysis

All MR images were jointly evaluated by two radiologists with 7 and 10 years of experience in neuroradiology, respectively. The assessment included T2-FLAIR, postcontrast 3D T1W, apparent diffusion coefficient (ADC) maps, susceptibility-weighted imaging (SWI), and cerebral blood volume (CBV) maps. In cases of uncertainty or disagreement, consensus was achieved with the guidance of a senior neuroradiologist with 28 years of experience. Throughout the image evaluation and measurement processes, the radiologists were blinded to the patients’ clinical and pathological data to minimize bias.

Lesion volume was defined as the entire contrast-enhancing tumor area on postcontrast 3D T1W images, including any central non-enhancing necrotic components. Both the lesion volume and the volume of the non-enhancing necrotic component were measured on these images. The necrosis ratio was calculated by dividing the volume of the non-enhancing necrotic component by the lesion volume, in line with previous methods reported by Yoo et al. [13] The peritumoral edema volume was measured on T2-FLAIR or T2W images, and the edema ratio was calculated similarly, by dividing the edema volume by the lesion volume [14].

The open-source, semi-automated software 3D Slicer^®^ was utilized for volumetric measurements [15]. After anonymization, the images were stored in Digital Imaging and Communications in Medicine (DICOM) format and imported into the software. The regions designated for volumetric measurements were semi-automatically segmented using the “Editor” module within the program, followed by manual corrections to refine the segmentation. The final measurements were then recorded.

Quantitative ADC measurements were performed using the Syngo.via^®^ (Siemens Healthineers, Erlangen, Germany) and Advantage Windows Workstation^®^ (Version 4.5, GE Healthcare, Fairfield, USA). For each lesion, three regions of interest (ROIs) were manually drawn in areas showing the most prominent diffusion restriction on visual inspection. The final ADC value was recorded as the average of these three measurements. ROI size ranged from 4 to 10 mm², depending on tumor size and morphology. ROIs were carefully placed to avoid necrotic, cystic, or hemorrhagic regions. ADC measurements could not be performed in 14 lesions (4.8%) due to insufficient lesion size (< 1 mm).

**Fig. 1:**
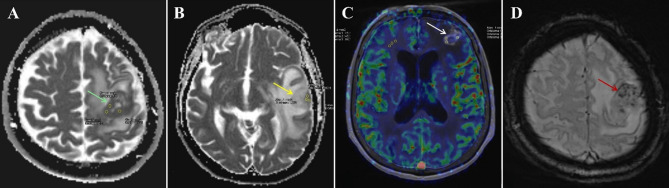
Green arrow (**A**) shows a SCLC brain metastasis with a mean ADC of 696 mm²/s. Yellow arrow (**B**) shows a NSCLC brain metastasis with a mean ADC of 994 mm²/s. White arrow (**C**) highlights a hypoperfused breast cancer brain metastasis with a rCBV of 0.948. Red highlights a malignant melanoma brain metastasis (**D**) with grade 3 ITSS score, including 12 dot-like susceptibilities

Perfusion MRI analysis was conducted using the Syngo.via^®^ workstation (Siemens Healthineers, Erlangen, Germany). Relative cerebral blood volume (rCBV) maps were generated from DSC perfusion data. To avoid necrotic, cystic, and large vascular areas, ROIs were manually placed in the most hyperperfused regions of the solid tumor component and in the contralateral normal-appearing white matter on the same axial slice. To minimize measurement variability, three ROIs were drawn within each lesion using a freehand technique, and the mean CBV value was calculated by averaging the three measurements. ROI dimensions ranged from 4 to 10 mm², adjusted according to the tumor’s size and morphology. If no hyperperfused region was identified within a lesion, measurements were taken from the non-perfused solid portion of the tumor. The rCBV ratio was then calculated using the formula: rCBV = CBV_Lesion​​_ / CBV_NormalWM_​ (Fig. 1).

Susceptibility signal changes within the lesions were assessed on SWI using a quantitative scoring system. The intratumoral susceptibility signal (ITSS) score was determined according to the number of dot-like or fine linear hypointense areas, which reflect intratumoral hemorrhage, microvascular proliferation, and neovascularization. Accordingly, the ITSS grades were defined as follows: Grade 0, No ITSS; Grade 1, 1–5 dot-like or fine linear ITSSs; Grade 2, 6–10 dot-like or fine linear ITSSs; and Grade 3: ≥11 dot-like or fine linear ITSSs in the continuous area within a tumor [16].

### Categorization of the patients

Patients were classified into five groups based on the origin of cancer: breast cancer, small cell lung cancer (SCLC), non-small cell lung cancer (NSCLC), malignant melanoma, and other cancers. The “other” category included gastrointestinal system adenocarcinomas, ovarian cancers, thymic carcinoma, renal cell carcinoma, parotid gland mucoepidermoid carcinoma, and oral cavity squamous cell carcinoma. Although the number of patients with malignant melanoma metastases was relatively small, they were categorized separately due to their distinct MRI characteristics.

Mortality statistics were last updated on December 18, 2024. As of this date, the mean follow-up period for surviving patients was 1446 (± 621) days.

### Statistical analysis

Statistical analyses were performed using IBM SPSS Statistics for Windows, Version 25.0 (IBM Corp., Armonk, NY, USA). A p-value < 0.05 was considered statistically significant. Numerical variables were expressed as median and interquartile range (IQR) for non-normally distributed data, and as mean ± standard deviation (SD) for normally distributed data.

In the lesion-based analysis, the associations between the primary cancer type and lesion volume, necrotic volume, necrosis ratio, peritumoral edema volume, edema ratio, and rCBV were evaluated using the Kruskal-Wallis test. Post hoc pairwise comparisons following significant results were conducted using the Dunn test with Bonferroni correction. The associations between the primary cancer type and ADC values and ITSS scores were assessed using one-way ANOVA followed by Bonferroni post hoc tests.

For visualization of data distributions, box-and-whisker plots were used for continuous variables including necrosis ratio, edema ratio, rCBV, and ADC.

Receiver Operating Characteristic (ROC) curve analysis was used to determine the optimal cut-off values for ADC and ITSS scores. Area under the curve (AUC), sensitivity, and specificity were calculated accordingly.

In the patient-based analysis, metastases were classified as solitary versus multiple. The relationship between metastasis multiplicity and the primary cancer type was evaluated using the Chi-square test. Lesions were also categorized based on size as small (≤ 0.5 cm³) versus others, and based on location as infratentorial versus supratentorial. For each classification, cancer subgroups were compared using Fisher’s Exact Test, and odds ratios (ORs) were calculated for significant findings.

## Results

Of the 125 patients included in the study, 52 (41.6%) were male and 73 (58.4%) were female. The mean age was 60 (± 10.9) years. A total of 291 metastatic lesions were identified, of which 203 (69.8%) were supratentorial, while 88 (30.2%) were infratentorial. Among the study population, 104 patients (83.2%) had died. The mean survival time was 774 (± 593) days.

Primary tumor types included breast cancer (*n* = 41, 32.8%), SCLC (*n* = 24, 19.2%), NSCLC (*n* = 35, 28%), malignant melanoma (*n* = 5, 4%), and other cancers (*n* = 20, 16%). A total of 291 metastatic lesions were recorded, with the highest counts observed in breast cancer (*n* = 111), NSCLC (*n* = 69), and SCLC (*n* = 54) (Table 3). Regarding the number of metastatic lesions per patient, 46 patients had a single metastasis, 33 had two, and 13 had three. The highest lesion count in a single patient was 11, observed in two individuals.

**Table 3:** Distribution of patients with brain metastases by primary cancer type

Primary Cancer	Number of patients (%)	Number of lesions (%)
Breast cancer	41 (32.8)	111 (38.1)
SCLC	24 (19.2)	54 (18.6)
NSCLC	35 (28)	69 (23.7)
Malignant melanoma	5 (4)	14 (4.8)
**Other cancers** (see below)	20 (16)	43 (14.8)
Ovarian cancers	7 (5.6)	14 (4.8)
Gastrointestinal system cancers	8 (6.4)	14 (4.8)
Parotid gland cancer	1 (0.8)	6 (2.1)
Squamous cell carcinoma	1 (0.8)	5 (1.7)
Thymic carcinoma	1 (0.8)	2 (0.7)
Renal cell carcinoma	2 (1.6)	2 (0.7)
Total	125 (100)	291 (100)

SCLC, Small Cell Lung Cancer; NSCLC, Non-Small Cell Lung Cancer

In the lesion-based analysis, the highest median lesion volume was observed in NSCLC, with 0.645 cm³ (0.144–2.287) (Table 4). However, there was no significant difference in lesion volume among the groups (*p* = 0.292). The highest median necrotic volume was also found in NSCLC, with 0.024 cm³ (0.000–0.248). The necrotic volume in NSCLC metastases was significantly higher than in breast cancer metastases (*p* = 0.029), but no significant differences were found among the other groups. The highest median necrosis ratio was recorded in NSCLC, with 0.070 cm³ (0.000–0.200). The necrosis ratio in NSCLC metastases was significantly higher than in breast cancer (*p* < 0.001) and SCLC metastases (*p* = 0.025). Additionally, metastases from other cancer types had a significantly higher necrosis ratio than breast cancer metastases (*p* = 0.037). No significant differences were observed in the necrosis ratio among the remaining groups. Regarding peritumoral edema, the highest median peritumoral edema volume was observed in NSCLC, with 2.534 cm³ (0.023–22.236) (Table 4). However, no significant difference was found among the groups (*p* = 0.058). The highest median edema ratio was recorded in NSCLC, with 5.030 cm³ (0.039–19.193). The edema ratio in malignant melanoma metastases was significantly higher than in breast cancer (*p* = 0.001) and SCLC metastases (*p* = 0.004). Additionally, NSCLC metastases exhibited a significantly higher edema ratio than breast cancer metastases (*p* = 0.011). No significant differences were observed in the edema ratio among the other groups.

**Table 4:** Differentiation of primary cancers in brain metastases with conventional MRI measurements

Cancer types	Tumor volume	Necrotic volume	Necrosis ratio	Peritumoral edema volume	Edema ratio
	Median (Q1-Q3)	Median (Q1-Q3)	Median (Q1-Q3)	Median (Q1-Q3)	Median (Q1-Q3)
Breast (n:111)	0.250 (0.056–0.941)	**0.000 (0.000-0.001)**	**0.000 (0.000-0.010)**	0.026 (0.001–2.085)	0.205 (0.014–3.366)
SCLC (n:54)	0.453 (0.113–1.770)	0.002 (0.000-0.098)	**0.020 (0.000-0.080)**	0.373 (0.005–8.129)	**0.915 (0.031–8.365)**
NSCLC (n:69)	0.645 (0.144–2.287)	**0.024 (0.000-0.248)**	**0.070 (0.000-0.200)**	2.534 (0.023–22.236)	**5.030 (0.039–19.193)**
Melanoma (n:14)	0.522 (0.098–1.325)	0.002 (0.001–0.088)	0.010 (0.000-0.053)	1.517 (0.002–8.062)	**1.468 (0.021–13.272)**
Other cancers (n:43)	0.334 (0.056–3.812)	0.008 (0.000-0.112)	**0.020 (0.000-0.140)**	1.609 (0.133–20.429)	3.015 (0.280-10.333)
Overall (n:291)	0.339 (0.086–1.654)	0.001 (0.000-0.062)	0.000 (0.000-0.080)	0.555 (0.003–6.793)	1.190 (0.024–8.469)

*Bold values indicate statistically significant differences in at least one comparison. Tumor volume, necrotic volume, and peritumoral edema volume are expressed in cubic centimeters (cm³). SCLC, Small Cell Lung Cancer; NSCLC, Non-Small Cell Lung Cancer; Q1–Q3, Interquartile Range

**Fig. 2:**
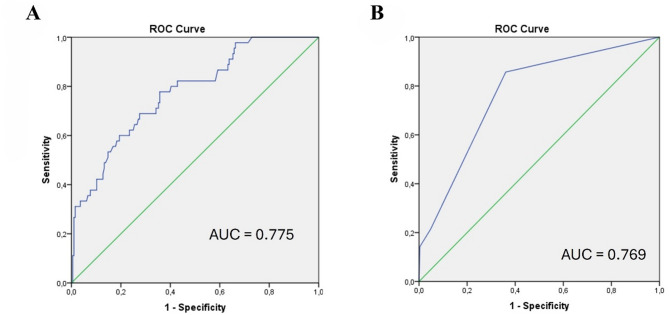
ROC curves for the differentiation of brain metastases using ADC values and ITSS scores. (**A**) Differentiation of NSCLC metastases based on ADC values (AUC = 0.775; optimal cut-off = 0.905 mm²/s; sensitivity = 77.8%, specificity = 64.3%). (**B**) Differentiation of malignant melanoma metastases based on ITSS score (AUC = 0.769; optimal cut-off = 1; sensitivity = 85.7%, specificity = 63.9%)

The lowest mean ADC value was observed in malignant melanoma, with 0.748 (± 0.222) mm²/s, while the highest mean ADC value was found in NSCLC, with 1.042 (± 0.193) mm²/s (Table 5). The mean ADC values in NSCLC metastases were significantly higher than in all other metastatic groups (all p-values < 0.001). However, no significant differences were found in ADC values among the remaining groups. The optimal cut-off value was determined as ≥ 0.905 mm²/s for distinguishing NSCLC metastases from other metastases using ADC. This threshold yielded a sensitivity of 77.8% and a specificity of 64.3%. The AUC was 0.775 (95% CI: 0.701–0.849) (Fig. 2).

The highest median rCBV value was observed in malignant melanoma, with 3.860 (2.218–4.793) (Table 5). Malignant melanoma and SCLC metastases had significantly higher rCBV values than breast cancer metastases (*p* = 0.02 and *p* = 0.047, respectively). No significant differences in rCBV values were found among the other groups.

**Table 5:** Comparison of ADC, rCBV, and ITSS values among different primary cancer types

Cancer types	ADC (mm²/s)	rCBV	ITSS score
	Mean (SD)	Median (Q1-Q3)	Mean (SD)
Breast (n:111)	0.877 (0.162)	**1.090 (0.870–1.875)**	**0.216 (0.475)**
SCLC (n:54)	0.825 (0.127)	**2.450 (1.500-4.645)**	**0.519 (0.666)**
NSCLC (n:69)	**1.042 (0.193)**	2.520 (1.520–2.720)	**0.613 (0.610)**
Melanoma (n:14)	0.748 (0.222)	**3.860 (2.218–4.793)**	**1.214 (0.893)**
Others (n:43)	0.858 (0.121)	1.495 (0.783–2.143)	0.500 (0.634)

*Bold values indicate statistically significant differences in at least one comparison. SCLC, Small Cell Lung Cancer; NSCLC, Non-Small Cell Lung Cancer; ADC, Apparent Diffusion Coefficient; rCBV, Relative Cerebral Blood Volume; ITSS, Intratumoral Susceptibility Signal; Q1–Q3, Interquartile Range; SD, Standard Deviation

**Fig. 3:**
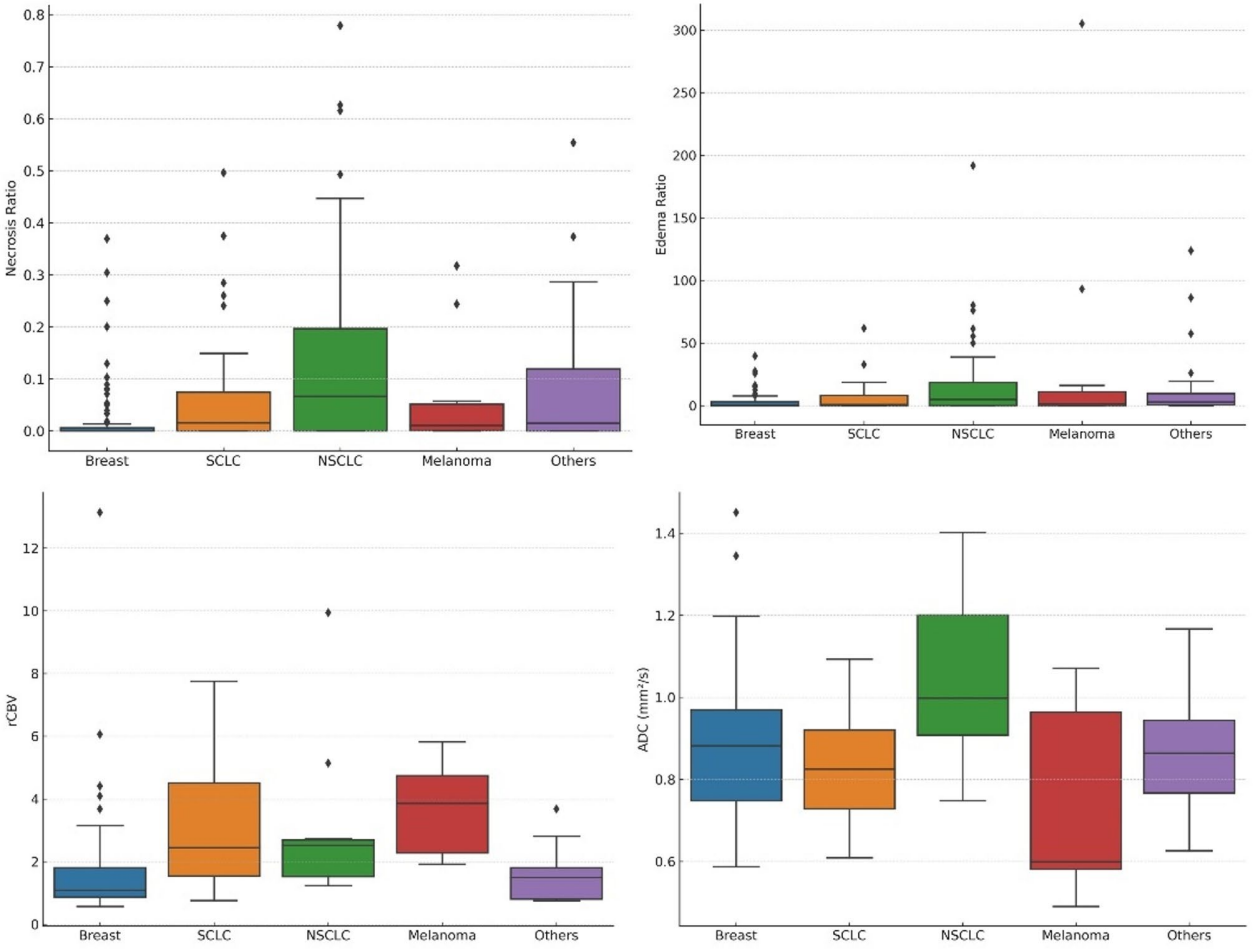
Box-and-whisker plots shows the distribution of necrosis ratio, edema ratio, ADC values, and rCBV values of brain metastases according to primary cancer origin

The distribution of necrosis ratio, edema ratio, rCBV, and ADC values across brain metastases from different primary cancers is shown in Fig. 3.

The highest mean ITSS score was found in malignant melanoma metastases, with 1.214 (± 0.893) while the lowest mean ITSS score was observed in breast cancer metastases, with 0.216 (± 0.475) (Table 5). The ITSS score in malignant melanoma metastases was significantly higher than in all other metastatic groups (p-values ranging from < 0.001 to 0.007). The optimal cut-off value was determined as ≥ 1 to differentiate malignant melanoma metastases with ITSS score. This threshold yielded a sensitivity of 85.7% and a specificity of 63.9%. The AUC was 0.769 (95% CI: 0.648–0.890) (Fig. 2). Additionally, breast cancer metastases had significantly lower ITSS scores than SCLC (*p* = 0.023) and NSCLC metastases (*p* < 0.001). No significant differences in ITSS scores were found among the remaining subgroups.

**Fig. 4:**
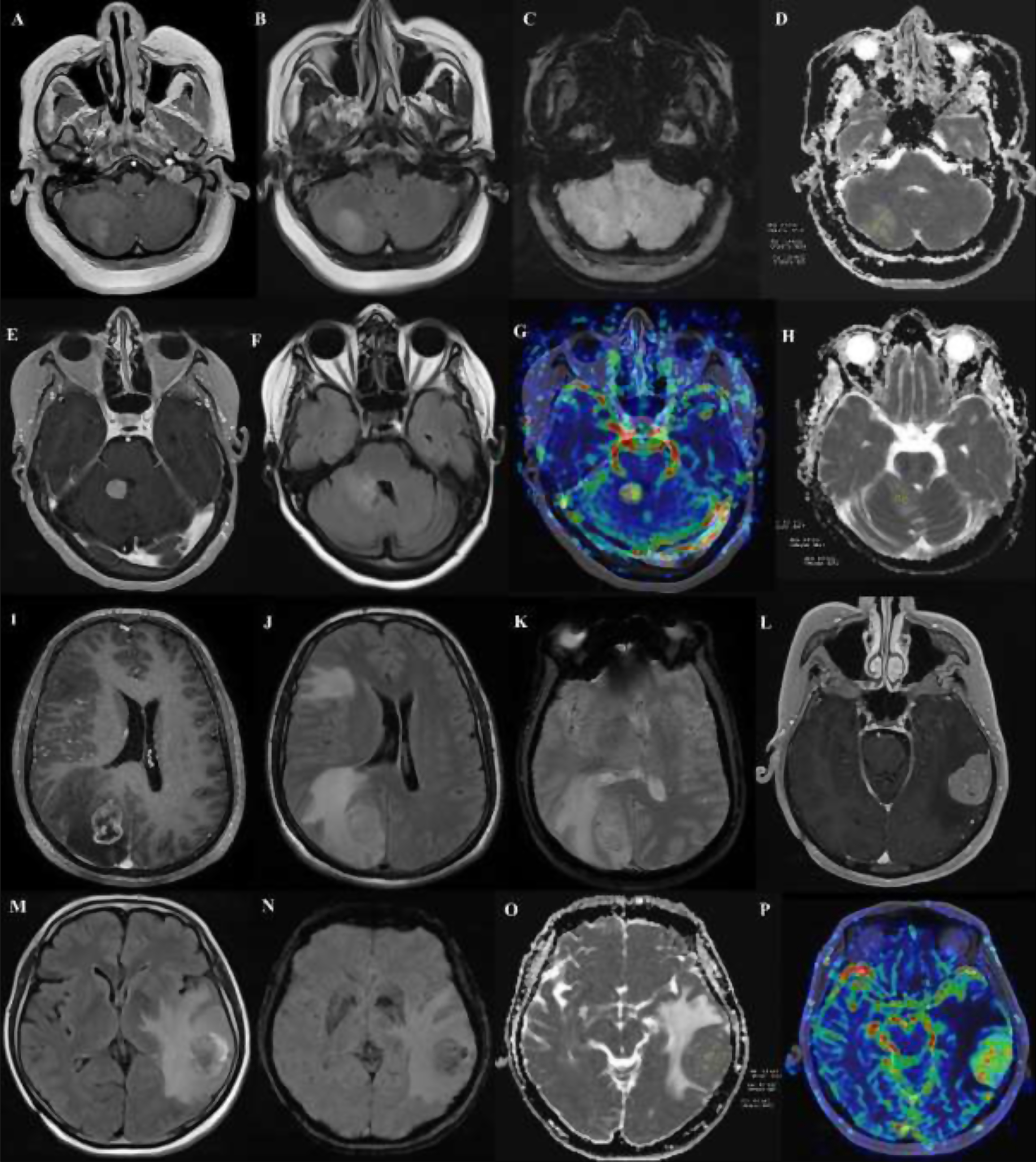
Representative multiparametric MRI findings of brain metastases from different primary tumors. (A–D) Breast cancer metastasis in the right cerebellar hemisphere. (**A**) Contrast-enhanced T1-weighted image demonstrates strong enhancement. (**B**) FLAIR image shows no significant peritumoral edema. (**C**) Susceptibility-weighted image reveals two punctate hypointense foci corresponding to an ITSS grade 1. (**D**) ADC map indicates a mean ADC value of 0.907 mm²/s. (**E**–**H**) Small cell lung cancer metastasis adjacent to the fourth ventricle. (**E**) Contrast-enhanced T1-weighted image shows an enhancing lesion. (**F**) FLAIR image shows minimal peritumoral edema. (**G**) Perfusion map demonstrates hyperperfusion, and (**H**) ADC map shows a mean ADC value of 0.829 mm²/s. (**I**–**K**) Non-small cell lung cancer metastasis with a central necrotic component. (**I**) Contrast-enhanced T1-weighted image shows irregular peripheral enhancement with central non-enhancing necrosis. (**J**) FLAIR image reveals extensive peritumoral edema. (**K**) Susceptibility-weighted image demonstrates two punctate hypointense signals (ITSS grade 1). (**L**–**P**) Malignant melanoma metastasis. (**L**) Contrast-enhanced T1-weighted image demonstrates vivid enhancement. (**M**) FLAIR image shows prominent peritumoral edema. (**N**) Susceptibility-weighted image reveals multiple hypointense foci (7–8 in total, ITSS grade 2). (**O**) ADC map shows a mean ADC value of 0.913 mm²/s. (**P**) Perfusion map demonstrates marked hyperperfusion

Representative multiparametric MRI examples of brain metastases from different primary tumors are presented in Fig. 4.

A total of 49 patients (39.2%) had solitary metastasis, with the following distribution: 17 breast cancer, 9 SCLC, 12 NSCLC, 2 malignant melanoma, and 9 other cancers. The mean number of metastatic lesions per patient was 3.12 (± 2.87) for breast cancer, 2.75 (± 2.87) for SCLC, 2.43 (± 1.56) for NSCLC, 2.4 (± 1.52) for malignant melanoma, and 2.15 (± 2.03) for other cancers. There was no statistically significant association between the number of metastases and tumor origin (*p* = 0.523). However, the presence of multiple metastases with a volume ≤ 0.5 cm³ was significantly associated with breast cancer metastases (*p* = 0.041, OR = 1.212, 95% CI: 1.008–1.458).

The distribution of supratentorial metastases was as follows: 64 breast cancer, 43 SCLC, 59 NSCLC, 14 malignant melanoma, and 23 other cancers. The distribution of infratentorial metastases was as follows: 47 breast cancer, 11 SCLC, 10 NSCLC, 0 malignant melanoma, and 20 other cancers. Infratentorial metastases were significantly more likely to originate from breast cancer (*p* = 0.001) or other cancer types (*p* = 0.018). The odds ratios were 2.490 and 2.302, respectively (Table 6).

**Table 6:** Comparative analysis of primary cancers with infratentorial metastases

Cancer type	Number of infratentorial metastasis	Odds Ratio	95% Confidence Interval	
			Lower	Upper
Breast	47	**2.490**	1.491	4.158
SCLC	11	0.532	0.260	1.088
NSCLC	10	0.313	0.151	0.646
Melanoma	0	0.0	-	-
Others	20	**2.302**	1.188	4.458

*Bold values indicate statistically significant differences. SCLC, Small Cell Lung Cancer; NSCLC, Non-Small Cell Lung Cancer

## Discussion

This study investigated whether multiparametric MRI features can be used to predict the primary origin of brain metastases at the time of diagnosis. Accurately identifying the primary tumor in cases of brain metastases remains a clinical challenge, particularly in patients in whom the primary site is unknown. While artificial intelligence algorithms have shown promise in this area, these tools are not yet integrated into daily clinical practice. Thus, simple and accessible MRI-based markers are still needed to support diagnostic decision-making. Our findings suggest that certain conventional MRI characteristics—such as lesion size, diffusion restriction, ITSS score, perfusion, and edema—vary systematically across different cancer types, offering clinically useful clues regarding tumor origin.

Our study demonstrated that NSCLC metastases exhibited significantly greater necrotic volume, necrosis ratio, and edema ratio compared to breast cancer metastases. The necrosis ratio was also higher in NSCLC than in SCLC, and melanoma metastases displayed greater edema compared to both breast cancer and SCLC. These imaging findings may be explained by underlying oncogenic mechanisms. Previous studies have suggested that increased oncogenic activity contributes to intratumoral necrosis [17], and that a higher degree of necrosis in brain metastases is associated with poorer prognosis [18]. These findings align with previous research. Yoo et al. reported that lung cancer metastases showed greater necrosis than other cancer types in a series of 141 craniotomy cases, suggesting that necrosis may correlate with tumor aggressiveness and could assist in identifying tumor origin [13]. Similarly, necrosis has been particularly noted in triple-negative breast cancer metastases [19]. With regard to peritumoral edema, Fabian et al. found that NSCLC metastases exhibited more edema than SCLC, and that edema thickness was correlated with lesion size [20]. This observation was supported by another study reporting a higher peritumoral edema ratio in NSCLC than in SCLC [14]. A comparative MRI study found that lung cancer metastases had larger volumes, more cystic changes, and more extensive edema compared to breast cancer metastases [21]. Tran et al. also observed increased peritumoral edema in malignant melanoma compared to NSCLC metastases [22]. Collectively, these results support the utility of necrosis and edema characteristics in differentiating the primary origin of brain metastases.

Our study demonstrated that NSCLC metastases had significantly higher ADC values than other primary cancer types. An optimal cut-off value of ≥ 0.905 mm²/s was identified for distinguishing NSCLC metastases, yielding a sensitivity of 77.8% and a specificity of 64.3%. This finding indicates that diffusion-weighted imaging may provide moderate discriminatory power in differentiating NSCLC metastases from other tumor origins. Previous studies support this observation. Meyer et al. reported that SCLC metastases exhibited the lowest ADC values (0.86 ± 0.27 mm²/s) among 948 brain metastases, while NSCLC metastases had higher ADC values (1.17 ± 0.49 mm²/s) [23]. Similarly, Müller et al. found mean ADC values of 0.68 ± 0.12 mm²/s in SCLC and 1.47 ± 0.31 mm²/s in NSCLC, with a proposed cut-off of 0.97 mm²/s for differentiation [24]. The elevated ADC values observed in NSCLC metastases may be partly explained by the presence of micronecrotic or cystic changes within the tumor, which can increase water mobility even in regions that appear solid on imaging. These characteristics may suggest a more heterogeneous and partially necrotic tumor architecture.

In our study, malignant melanoma and SCLC metastases exhibited significantly higher rCBV values than breast cancer metastases, suggesting increased perfusion in these tumor types.

Previous studies have primarily focused on the role of perfusion MRI in distinguishing glial tumors from other conditions such as radiation necrosis, lymphoma, and metastases. A limited number of studies have explored the potential of perfusion imaging in differentiating brain metastases according to their primary origin, but these have often been conducted on relatively small patient cohorts. Askaner et al. reported that while higher perilesional CBV values could differentiate glial tumors from brain metastases, CBV was not effective in identifying the primary origin of metastases [25]. In contrast, two separate studies have reported that melanoma brain metastases tend to exhibit higher perfusion values, suggesting distinct vascular characteristics associated with this tumor type [26, 27]. The increased rCBV observed in melanoma and SCLC metastases may be related to tumor-specific angiogenic activity or microvascular alterations, possibly driven by underlying molecular mechanisms. However, further studies are needed to clarify these associations.

In our study, malignant melanoma metastases exhibited significantly higher ITSS compared to all other tumor types. Additionally, breast cancer metastases showed lower ITSS than both SCLC and NSCLC metastases. These findings suggest that intratumoral susceptibility signals may vary depending on the primary origin of brain metastases. Previous studies have associated increased ITSS with higher vascularity, neoangiogenesis, and microhemorrhage, and have proposed it as a marker of tumor aggressiveness in glioma grading [16, 28]. In the study by Radbruch et al., malignant melanoma metastases demonstrated significantly higher ITSS than breast and lung metastases, with a reported AUC of 0.96 for this distinction on SWI [29]. Another study noted that intratumoral hemorrhage is rarely observed in micrometastases smaller than 0.1 cm, and that malignant melanoma metastases tended to show more hemorrhage than breast cancer metastases, likely due to their generally larger size [30]. Moreover, no significant differences in ITSS were observed between melanotic and amelanotic melanoma subtypes, suggesting that susceptibility signals in SWI primarily reflect hemorrhagic or paramagnetic content rather than melanin [31]. In our study, ITSS was scored according to the criteria defined by Park et al. [16]. To the best of our knowledge, there is no prior study in the English-language literature that has applied the ITSS scoring system for distinguishing brain metastases by primary tumor type. In this regard, our study provides an additional contribution to the existing body of literature.

In our study, multiple brain metastases were observed in 61.2% of patients. Breast cancer and SCLC cases had the highest mean number of metastases per patient. The smallest metastases were detected in breast cancer patients. However, no statistically significant difference in lesion size was observed across primary tumor types. Nonetheless, multiple metastases with a volume ≤ 0.5 cm³ (≤ 1 cm in a single axis) were significantly associated with breast cancer (*p* = 0.010). Additionally, infratentorial metastases were more frequently associated with breast cancer and with tumors in the “other” group (e.g., gastrointestinal and ovarian cancers), while no infratentorial melanoma metastases were detected in our sample. Our findings are generally consistent with prior studies. Lung cancer and melanoma metastases have been reported to frequently present as multiple brain lesions [32]. It has also been suggested that up to 78% of breast cancer metastases may develop as multiple parenchymal lesions [33]. In a large study of 3313 intra-axial metastases, Schroeder et al. reported that breast cancer patients had the highest median number of brain metastases per patient, and the smallest metastases were also observed in this group. They also noted a greater frequency of cerebellar metastases in breast and gastrointestinal cancers [34]. Similarly, Dou et al. found that infratentorial metastases were commonly associated with NSCLC, breast, and colorectal cancer, but were notably absent in malignant melanoma [35]. In the literature, breast cancer metastases have been reported to be smaller than lung cancer metastases [36]. In another study evaluating 314 metastases and comparing breast and lung cancer brain metastases, breast cancer lesions were found to be smaller on average, although the difference was not statistically significant [21]. Our findings suggest that breast cancer brain metastases tend to present as small, multiple, and infratentorial lesions. Therefore, to avoid missing small lesions, all brain regions should be carefully evaluated when a metastasis is identified in breast cancer patients. Overlooking such lesions due to their size or multiplicity may lead to underestimation of disease burden and negatively affect treatment planning.

This study has several limitations. Firstly, its retrospective, single-center design may introduce selection bias and limit generalizability, though image evaluators were blinded to tumor origin. The proportion of lung cancer metastases was relatively low, likely due to the presence of another specialized treatment center in our region. Imaging was performed on two different 1.5 Tesla scanners; future multi-center studies including varied magnetic field strengths and manufacturers would improve external validity. In DSC perfusion analyses, lesions with major hemorrhage were excluded and ROI placement avoided visible hemorrhagic areas, though subtle effects from microscopic bleeding in high ITSS lesions may still have occurred. Another limitation is that histopathological confirmation of metastatic brain lesions was not available for all cases. Diagnoses were based on the pathological confirmation of the primary tumor, comprehensive clinical evaluation, and systematic whole-body screening (including PET-CT, endoscopy-colonoscopy, and thoracoabdominal CT) to exclude other primary malignancies. We believe that assuming a different primary origin in patients with a known primary cancer and negative systemic screening is clinically less likely. However, we acknowledge that the possibility of misclassification cannot be entirely excluded. Additionally, variations in the timing of brain MRI, ranging from incidental detection during follow-up to symptom-prompted imaging, may have influenced the number of detected metastases. Moreover, prior treatments for the primary cancer, which were not systematically assessed in this study, may have influenced the timing and imaging characteristics of brain metastases. Another limitation is that while our categorization into five main groups provides broad insights, the limited sample sizes for specific types like malignant melanoma (*n* = 5) and ovarian cancers (*n* = 7) may affect the statistical power for these less frequent subgroups. Additionally, inherent heterogeneity within the “other cancers” category and the lack of subgroup analyses based on specific biological characteristics, such as breast cancer receptor status, molecular or genetic mutations (e.g., EGFR, ALK, BRAF) and immunological markers (e.g., PD-1/PD-L1), meant that further distinctions could not be made. Such data was not systematically available for all patients in our retrospective dataset, precluding reliable molecular- or immunology-based subgroup analyses. Future, more focused, and preferably prospective studies are needed to explore the relationship between these molecular/immune profiles and multiparametric MRI characteristics in greater detail. Despite these limitations, our study contributes valuable insights by systematically evaluating conventional multiparametric MRI characteristics for differentiating primary cancer origins in brain metastases, offering clinically accessible markers that can guide diagnostic decision-making in complex clinical scenarios.

## Conclusion

Our study demonstrates that brain metastases exhibit identifiable differences in multiparametric MRI characteristics based on their primary cancer origin. Specifically, breast cancer metastases tend to be smaller, multiple, and infratentorial. NSCLC metastases are typically more necrotic with higher ADC values, while melanoma metastases consistently show higher intratumoral susceptibility. These distinct imaging features provide valuable diagnostic clues that can aid in the differential diagnosis of primary cancer types in patients with brain metastases.

## Data Availability

The datasets used and/or analyzed during the current study are available from the corresponding author on reasonable request.

## Abbreviations

ADC: Apparent Diffusion Coefficient
AUC: Area Under the Curve
BM: Brain Metastases
CBV: Cerebral Blood Volume
CI: Confidence Interval
CT: Computed Tomography
DICOM: Digital Imaging and Communications in Medicine
DSC: Dynamic Susceptibility Contrast
DWI: Diffusion-Weighted Imaging
FLAIR: Fluid-Attenuated Inversion Recovery
FOV: Field of View
GRE: EPI-Gradient-Echo Echo-Planar Imaging
IQR: Interquartile Range
ITSS: Intratumoral Susceptibility Signal
MRI: Magnetic Resonance Imaging
NSCLC: Non-Small Cell Lung Cancer
OR: Odds Ratio
PET: CT-Positron Emission Computed Tomography
rCBV: Relative Cerebral Blood Volume
ROC: Receiver Operating Characteristic
ROI: Region of Interest
SCLC: Small Cell Lung Cancer
SD: Standard Deviation
SWAN: Susceptibility Weighted Angiography
SWI: Susceptibility-Weighted Imaging
TE: Echo Time
TI: Inversion Time
TR: Repetition Time
T1W: T1-Weighted
T1W: 3D-Three-Dimensional T1-Weighted
T2W: T2-Weighted
